# Forecasted Trends of the New COVID-19 Epidemic Due to the Omicron Variant in Thailand, 2022

**DOI:** 10.3390/vaccines10071024

**Published:** 2022-06-27

**Authors:** Rapeepong Suphanchaimat, Pard Teekasap, Natthaprang Nittayasoot, Mathudara Phaiyarom, Nisachol Cetthakrikul

**Affiliations:** 1 Division of Epidemiology, Department of Disease Control, Ministry of Public Health, Nonthaburi 11000, Thailand; rapeepong@ihpp.thaigov.net (R.S.); n.natthaprang@gmail.com (N.N.); 2 International Health Policy Program, Ministry of Public Health, Nonthaburi 11000, Thailand; p.mathudara@gmail.com; 3 Faculty of Business and Technology, Stamford International University, Bangkok 10250, Thailand; pteekasap@gmail.com

**Keywords:** COVID-19, vaccine, SARS-CoV-2, Omicron

## Abstract

Thailand is among many countries severely affected by COVID-19 since the beginning of the global pandemic. Thus, a deliberate planning of health care resource allocation against health care demand in light of the new SARS-CoV-2 variant, Omicron, is crucial. This study aims to forecast the trends in COVID-19 cases and deaths from the Omicron variant in Thailand. We used a compartmental susceptible-exposed-infectious-recovered model combined with a system dynamics model. We developed four scenarios with differing values of the reproduction number (R) and vaccination rates. In the most pessimistic scenario (R = 7.5 and base vaccination rate), the number of incident cases reached a peak of 49,523 (95% CI: 20,599 to 99,362) by day 73, and the peak daily deaths grew to 270 by day 50. The predicted cumulative cases and deaths at the end of the wave were approximately 3.7 million and 22,000, respectively. In the most optimistic assumption (R = 4.5 and speedy vaccination rate), the peak incident cases was about one third the cases in the pessimistic assumption (15,650, 95% CI: 12,688 to 17,603). In the coming months, Thailand may face a new wave of the COVID-19 epidemic due to the Omicron variant. The case toll due to the Omicron wave is likely to outnumber the earlier Delta wave, but the death toll is proportionately lower. Vaccination campaigns for the booster dose should be expedited to prevent severe illnesses and deaths in the population.

## 1. Introduction

Over the past 2–3 years, the world has recognized the coronavirus disease 2019 (COVID-19) pandemic as one of the most serious health threats in human history. The disease is caused by severe acute respiratory syndrome coronavirus-2 (SARS-CoV-2), which is transmitted by direct contact with droplets containing pathogens and indirect contact with contaminated surfaces [1,2]. The first reported case of COVID-19 was found in China, but then the disease spread rapidly throughout the world and became a global pandemic [3]. In January 2022, the global case toll had reached almost 300 million, with approximately 5.5 million accumulated deaths [4].

A SARS-CoV-2 variant with a significant genetic change from the original strain that demonstrated increased transmissibility and severity and showed evidence of immunity escaped characterized by the World Health Organization (WHO) as a variant of concern (VOC) [5,6]. During the second and the third quarters of 2021, the world was severely hit by the Delta variant, which was first detected in India [7,8]. By late 2021, while the world was hoping to see a promising end to the pandemic as global incident cases gradually subsided, another VOC, the Omicron variant, was reported. It was believed to have numerous mutations, with the potential to increase transmissibility compared with prior variants, and to partially escape infection- or vaccine-induced immunity [9,10,11].

Thailand is among many countries that have been critically affected by the COVID-19 pandemic. The first COVID-19 wave in Thailand occurred during March–May 2020 due to super-spreading events from a boxing stadium and a nightlife hotspot in Bangkok downtown [12]. The second wave originated from a cluster of cases in a shrimp market in the inner city of Samut Sakhon and lasted between December 2020 and February 2021 [12,13]. The third wave was mostly caused by the Alpha variant in April 2021, followed by the fourth wave beginning in June 2021 due to extensive local transmission of the Delta variant [13]. During that time, the Thai government implemented a lockdown policy as a pre-emptive measure to avoid the collapse of the health care system, and COVID-19 vaccines were rapidly rolled out. By early December, the volume of Thais receiving at least one shot of the COVID-19 vaccine numbered about 70% of the total population, the benchmark believed to make the country achieve herd immunity [14].

In December 2021, the Thai Ministry of Public Health (MOPH) declared the discovery of the first imported case of the Omicron variant, and the local transmission of the Omicron variant was confirmed. This situation caused significant concern for the government that the Thai health care system might risk collapsing as during the Delta pandemic.

This study therefore aims to forecast the trends of new cases as well as the death toll and use of health resources for severe cases given the advent of the Omicron variant in Thailand. We hope that the findings of this study will help aid policy decisions for optimal preparation of the country’s health care system in response to the coming pandemic.

## 2. Materials and Methods

### 2.1. Study Design

A secondary data analysis was employed. Most parameters used in this study were acquired from the internal database of the Department of Disease Control (DDC) and the Department of Medical Services (DMS). Some basic parameters such as incubation period and infectious duration were obtained from international literature. Parameters reflecting the Thai health care system performance were obtained from expert opinions and model adjustment. The forecasting duration was 120 days. It is worth noting that as the Omicron variant is quite new to the world, some variant-specific parameters were not available at the time of writing. We therefore adopted the parameters specific to the Delta variant instead.

### 2.2. Model Framework

**Figure 1:**
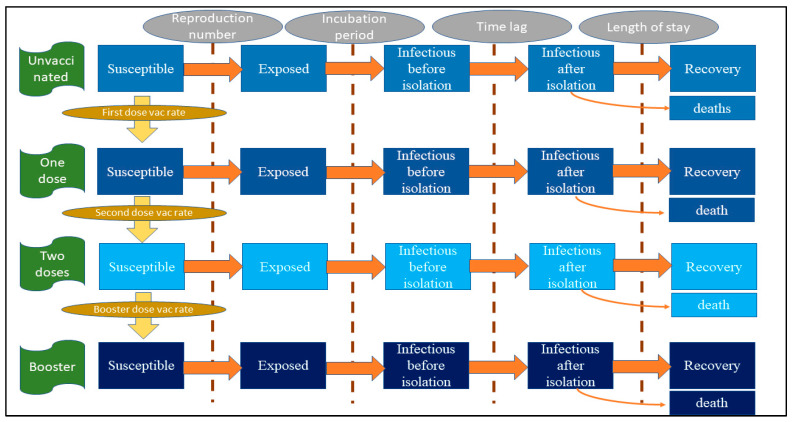
Model framework.

We employed a compartmental susceptible-exposed-infectious-recovered (SEIR) model and the system dynamics (SD) model to frame the analysis [15,16]. The SEIR model was commonly used to explain the epidemic force in many diseases (such as HIV, influenza, and tuberculosis) long before the COVID-19 pandemic [17,18,19]. The model simply explains the rate of change of the unit of interest from one stage to another. This concept is also in line with the idea of the stock and flow diagram, which is often applied in the SD model. The simplified model framework is demonstrated in Figure 1. We divided the entire Thai population into four groups based on the vaccination profile: (i) the unvaccinated, (ii) the one-dose, (iii) the two-dose, and (iv) the booster (receiving at least three shots of vaccine). In each group, we categorized the population into five subcategories according to the infection status: (i) the susceptible, (ii) the exposed, (iii) the infectious before isolation, (iv) the infectious after isolation, and (v) the recovered. The speed of transfer from the susceptible group to the exposed group was mainly influenced by the reproduction number (R) [20]. The transition from the exposed group to the infectious group depended on the incubation period. We adapted the traditional SEIR model by splitting the infectious group into before isolation and after isolation. The reason behind this is that once admitted to a hospital, an infected person would be isolated by the hospital protocol (supposing no nosocomial infection). The length of stay (LOS) in a hospital influenced the speed of recovery. Among the admitted patients, the prevalence of intubated cases attracted the attention of policy makers the most. This is because the volume of intubated cases represents the reserve capacity of intensive care, while asymptomatic or mild cases are allowed to be isolated at home or in the community according to the current MOPH protocol [21]. We further assumed that some of the intubated cases later died and that no deaths occurred without intubation. An unvaccinated susceptible person encountered two paths, either becoming exposed to the disease or remaining as a susceptible person and receiving the first vaccine shot, which depended on the vaccination rate in the entire population. The same concept also applied for the one-dose, the two-dose, and the booster groups.

### 2.3. Model Assumptions, Parameters and the Formula

The model was governed by the following assumptions. First, the population was homogenously mixed, allowing for inferring that all susceptible individuals were subject to infection (with varying probabilities conditional on vaccination status). This assumption coincided with present evidence that pointed to the immune–escape property of the Omicron variant [22,23].

Second, in most pandemics, the exact number of initial infectees could hardly be identified. We proposed that the volume of infectees equaled 10,000, about threefold the daily incident cases at the time of this writing. This assumption corresponded with the experience of the DDC outbreak investigators that when a super-spreading event was notified, approximately three to four generations of infection had already passed by.

Third, there existed some degree of underreporting for asymptomatic and mildly symptomatic infectees. The literature suggested that underreporting was common during the surge of the outbreak [24]. The field operations of the Rural Doctor Society of Thailand in mid-2020 affirmed that from an active screening, over 13% of people in high-density communities in Bangkok were found infected with COVID-19 but their records were not present in the official MOPH infectee list [16]. However, we postulated that there was no underreporting of intubated cases and deaths.

Fourth, recent evidence affirmed that the Omicron variant is transmitted more easily and faster than the Delta variant. Based on the model calibration against the incident cases during the peak of the Delta epidemic (July–December 2021), we found the value of 1.38 best represented the R for the entire population. We further proposed that if the Omicron variant caused another epidemic wave in Thailand, its R value would be about 3.1–5.4 times larger than that of the Delta variant [25,26]. These figures were later used to construct the model scenarios.

Fifth, in reality, the R did not remain constant over time due to social adaptability and various social measures. We postulated that it took 15 days for the R of the Omicron variant to climb from the current R in Thailand at the time of this writing (0.86) to reach the set value (fourth assumption) and that then it naturally dropped by two points within 60 days later.

Fifth, the R was influenced by two key factors: (i) the vaccine effectiveness (VE) against any infection and (ii) the contact rate of the people in the society. The COVID-19 vaccine not only acted on the probability of susceptibility to being exposed but also altered the severity profile of the infectious compartment (reducing the probability of becoming severe cases or deaths). Since no officially published report of the VE against the Omicron infection in Thailand had come out yet, we used the VE of the viral-vector vaccines in the UK instead (also the same vaccine type widely administered in Thailand) [27].

Sixth, we used the vaccination rate as of late 2021 as a base vaccination rate in the population and assumed that this remained unchanged throughout the study course. We touched upon the vaccination rate again in the later section, “Model scenarios and interested outcomes”. The COVID-19 vaccines were administered only when an individual lay in the susceptible state.

Lastly, and linked to the fifth assumption, the contact rate of individuals depended on the magnitude of the outbreak. Social measures and individual protective behaviors become stricter when the volume of incident cases enlarges. This idea concurred with the fact that the mobility trend (using public transport use as a proxy) of Thai individuals diminished by 68% during the Delta epidemic compared with the pre-COVID-19 era. By December 2021, when the Delta epidemic declined, the mobility trend declined by 25% relative to before 2020 [28]. We therefore added a parameter reflecting the effectiveness of social measures in the model.

We used Microsoft Excel and Stella 2.0 (number: 251-401-786-859) for model execution. Table 1 and Table 2 exhibit the important parameters and formulas of the model.

### 2.4. Model Scenarios and Interested Outcomes

We focused on the following outcomes: (i) daily reported incident cases, (ii) daily deaths, (iii) prevalent intubated cases (requiring invasive ventilator), (iv) cumulative case toll and (v) cumulative death toll. These outcomes were commonly used by the MOPH to gauge the health care burden. We constructed the interested scenarios by varying the values of R (highly transmitted [R = 4.3] versus very highly transmitted [R = 7.5]) and the vaccination rate (base pace (as shown in Table 1) versus speedy pace [three times faster than the base pace]) (Table 3). Scenario 1 was most pessimistic, whereas scenario 4 was most optimistic. Scenarios 2–3 were between the two ends. The model was run 100 times for each scenario, using infectious duration and incubation period as the sensitivity parameters.

## 3. Results

**Figure 2:**
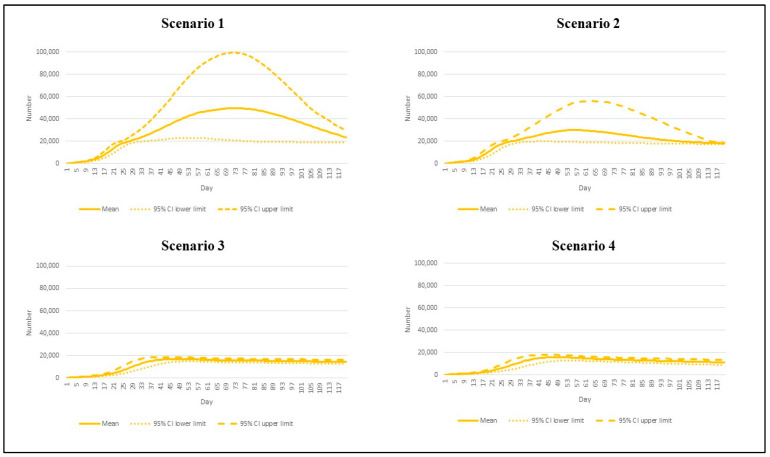
The daily incident cases by different epidemic scenarios.

We first presented the number of estimated daily reported cases in Figure 2. In the most pessimistic scenario (scenario 1), the peak daily incident cases exceeded other scenarios. The incident cases reached 49,523 per day by day 73 (95% CI: 20,599 to 99,362). Scenario 2, where the vaccination rate was sped up but the R remained high (7.5), saw the peak incident cases of 30,025 (95% CI: 19,358 to 54,317). With the R dipping down to 4.3 in scenarios 3 and 4, we found the peaks of the daily new cases at 16,889 (95% CI: 14,644 to 18,437) and 15,650 (95% CI: 12,688 to 17,603), respectively, by about day 50.

**Figure 3:**
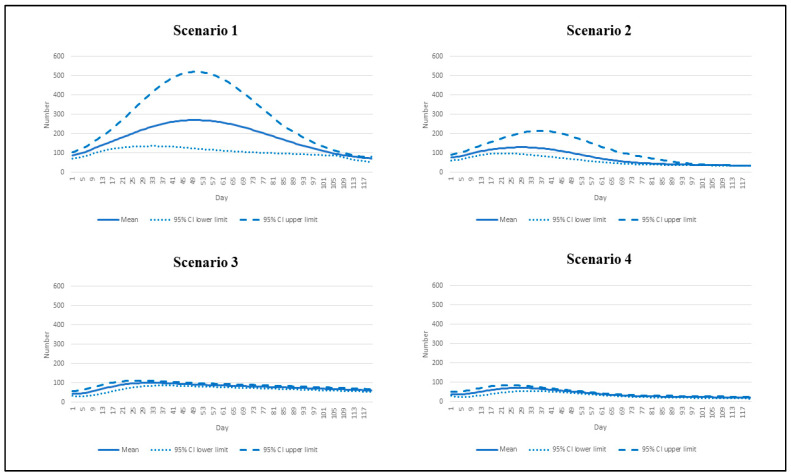
Daily deaths by different epidemic scenarios.

For the daily deaths, the number exceeded 100 by day 5 and reached the peak at 270 (95% CI: 126 to 518) by about day 50 in scenario 1. The second-highest toll presented in scenario 2 with a peak of 129 deaths by approximately day 30 (95% CI: 91 to 211). Scenarios 3 and 4 demonstrated almost the same pattern over the course of the analysis. Scenario 4 showed the smallest number of peak daily deaths relative to the other scenarios (72 deaths by day 28, 95% CI: 54 to 84) (Figure 3).

**Figure 4:**
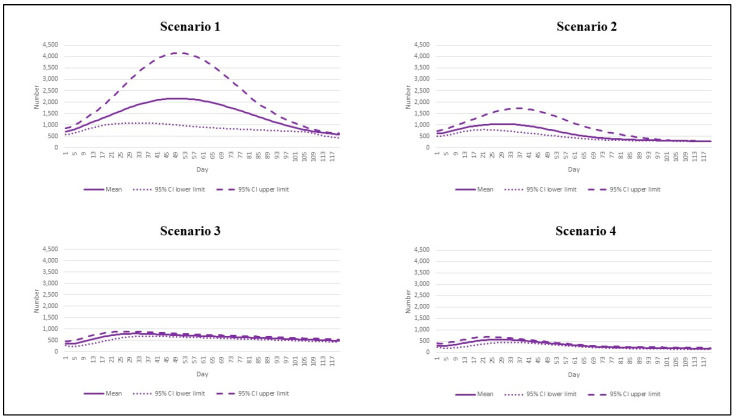
The prevalent intubated cases by different epidemic scenarios.

The prevalence of intubated cases followed the same pattern as the daily deaths. Scenario 1 saw the highest number of prevalent intubated cases (2161 cases by 50, 95% CI: 987 to 4161). Scenario 2 exhibited the second-highest peak, following scenario 1. The peak number reached 1035 by day 30 (95% CI: 743 to 1658). In scenario 3, the peak dropped to 800 during days 31–32 (95% CI: 675 to 888), and this further dipped to 572 in scenario 4 (95% CI interval: 429 to 675) (Figure 4).

**Figure 5:**
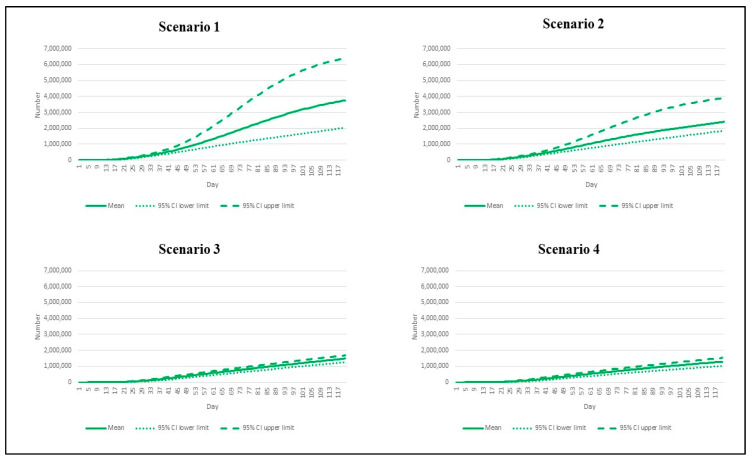
Cumulative case tolls by different epidemic scenarios.

The mean of cumulative incident cases by day 120 accounted for approximately 3.7 million (95% CI: 2,035,674 to 6,401,118). This number was about threefold higher than the mean cumulative cases in scenario 4 (about 1.3 million, 95% CI: 1,016,258 to 1,521,434). The mean cumulative case toll in scenarios 2–3 was between 1.5 million and 2.4 million (Figure 5).

**Figure 6:**
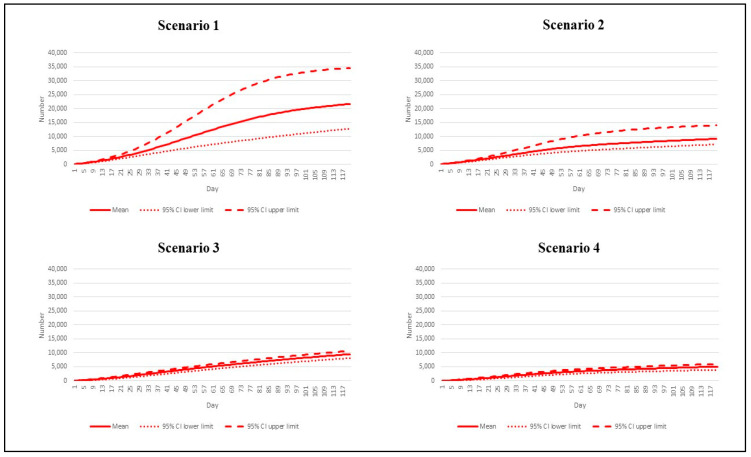
Cumulative death tolls by different epidemic scenarios.

Like other indicators, by day 120, scenario 1 presented the largest mean cumulative deaths at 21,649 (95% CI: 12,812 to 34,566). The mean death toll in scenario 4 was 4,996 (95% CI: 3922–5948), just a quarter of scenario 1’s figure. Scenarios 2–3 showed almost the same volume of cumulative deaths, between 9163 and 9467, by the end of the study period (Figure 6).

## 4. Discussion

Overall, the results suggest that the advent of the Omicron variant in Thailand may lead to a sharp increase in SARS-CoV-2 transmission. In the pessimistic scenario where the Omicron variant is very highly transmissible and the vaccination rate continues at the current pace, the peak incident cases may far exceed the 2021 Delta wave peak (~50,000 versus 22,000 cases a day). However, daily deaths due to Omicron epidemic may not outstrip the deaths in the Delta wave (~270 versus 300 deaths during the peak).

Our findings are in line with the current Omicron epidemic in numerous countries. The US has just faced a record high of daily new cases. About one million cases hit the US within a day in the beginning week of 2022 as hospitalization records approached 123,000, almost on par with the record high [32]. However, deaths remained fairly stable at about 1400 a day, well below the previous epidemic [32]. The UK also experienced a rapid surge of the people infected with the Omicron variant, bringing the UK total cases during the first week of 2022 to almost 1.3 million, about 30% higher than the week before [33]. The fourth epidemic wave in South Africa, caused by the Omicron variant, exhibited a peak of about 23,000 daily new cases, about one fifth greater than the earlier peak in June 2021 [34].

Concerning policy implication, this research informs us that although with a pessimistic assumption, the death toll may not exceed the prior peak, a large number of cases should not be overlooked because the volume of severe cases could skyrocket in proportion to the case toll. In the worst scenario, the need for ventilators almost reaches 2500 people per day by day 50. Such a demand has already surpassed the nationwide ventilator reserve, which is set at about 2200. Note that the reserve is for all patients needing invasive ventilators, not only COVID-19 cases. During the peak of the Delta wave, the prevalent intubated cases soared to about 1200 a day. With this demand, the Thai health care system was already stretched as most hospitals were in dire lack of intensive care beds and ventilators [35]. Another consideration is that the consumption of invasive ventilators by COVID-19 cases partly means a compromise in the quality of care for other patients [36,37].

This study also affirms the value of vaccinations to fight the Omicron variant. The peak of daily cases in scenario 2, in which we assumed very high transmissibility of the Omicron variant, is approximately 40% lower than the peak in scenario 1. Note that the benefit of vaccination diminishes when comparing scenario 4 with scenario 3 as the force of the epidemic was presumed to be less severe. This finding aligns with the latest evidence from the Canada and the UK, which suggests that despite the immune-escape characteristic of the Omicron variant, the third dose of COVID-19 vaccine provides some protection at least in the immediate term [38,39]. The bottom line is that now Thailand is witnessing vaccine administration at about 320,000 doses a day, still far lower than the maximum capacity of 1,000,000 doses a day, the figure set by the government as a campaign to beat the Delta wave last year [40]. Policy-wise, policy makers should consider the results from this study as part of the input for policy decision-making, especially for health resource planning. While recognizing context difference between countries, the findings from this study are still worth learning in terms of lessons from other nations. Some key lessons include that accelerating vaccine roll-out and maintaining a high degree of social measures can help alleviate the case burden. Additionally, from a technical perspective, academics from other countries could apply a compartmental model in combination with a SD model, as in this study, to estimate the volumes of cases and deaths from COVID-19 in their own setting.

This study is subject to some limitations. First, we did not account for the granular differences in the transmission rate of the Omicron variant across provinces. Moreover, we did not consider the impact of localized interventions. Second, we postulated a constant proportion of severe cases over the course of analysis. This may not be the case as the case fatality rate and the proportion of patients encountering severe conditions may increase during the period of high strain on health care facilities. Third, the analysis was surrounded by numerous uncertainties of the assumptions and the parameters. Knowledge of the Omicron variant, both in virology and in public health, is yet to be confirmed. Moreover, it is possible that new subvariants may emerge as genetic mutation is a common phenomenon of the virus. An obvious example is the advent of sub-lineages BA.4 and BA.5 of the omicron variant, which gained attention in the field of public health after the study was completed. The latest evidence showed moderate immunity escape of sub-lineages BA.5 and BA.5. However, there were no significant differences in terms of observed clinical severity and hospitalization rate between BA.4 and BA.5 and other Omicron sub-lineages [41]. If BA.4 and BA.5 sub-lineages were circulating in the study population, the forecasted case toll would be underestimated. Therefore, regular updating on the basic biological knowledge of the virus should be pursued as this will make the finding more robust and better fit to the current situation. Lastly, like in many other modelling studies, we de-constructed the force of the epidemic into various components, including contact rate, immunization rate, and vaccine effectiveness, to identify public health implications. However, we realized that it is extremely difficult to disentangle the biological characteristics of the virus from the social interventions or to exactly ascribe the epidemic phenomenon to a particular determinant. A clear example is the R, which is not a biological constant of a pathogen as it is also influenced by many other determinants, such as environmental conditions and behaviors of the infectees. Thus, the results should be interpreted with caution.

## 5. Conclusions

Under the most pessimistic presumption, the Omicron epidemic in Thailand may cause a peak of daily incident cases of about 50,000 by day 73. The peak daily death toll may reach 270 by day 50, corresponding with the peak prevalent intubated cases at about 2200. The acceleration of vaccine rollout will push down daily cases by up to 40% and the death toll by up to 55%. These figures should be used as input for the planning of health care resource allocation, especially intensive-care beds and respiratory ventilators, to meet the high demand of care given the coming Omicron epidemic. A national campaign to expedite vaccination rollout alongside an emphasis on the importance of individuals keeping a high protective guard is recommended.

## Figures and Tables

**Table 1:** List of essential parameters.

Parameters	Unit	Value	Reference (Note)
Reproduction number	Unitless	4.3–7.5	Ito et al. [25], Head and van Elsland [26] (3.1–5.4 times greater than the Delta epidemic in Thailand in 2020)
Population	Persons	66.2 × 10^6^	National Statistical Office of Thailand [29] (assume homogenous mixing)
Mean infectious duration	Days	4.6	Hart et al. [30] (assume gamma distribution with scale parameter of 0.03 and shape parameter of 165.9—same as the Delta variant)
Mean incubation period	Days	3.2	Helmsdal et al. [31] (assume gamma distribution with scale parameter of 0.01 and shape parameter of 302.7—shorter than the Delta variant)
Time lag from being infected to isolation	Days	5	Model calibration (assume same as the Delta epidemic in Thailand in 2020)
Initial number of infectees	Persons	10,000	Model calibration (assume fourfold greater than the present incident cases)
Initial proportion of unvaccinated population	Unitless	0.26	Internal database of the Department of Disease Control
Initial proportion of one-dose vaccinees	Unitless	0.09	Internal database of the Department of Disease Control
Initial proportion of two-dose vaccinees	Unitless	0.58	Internal database of the Department of Disease Control
Initial proportion booster-dose vaccinees	Unitless	0.06	Internal database of the Department of Disease Control
First-dose vaccination base rate	Persons/day	66,200	Internal database of the Department of Disease Control (assume equaling the rate of booster dose)
Second-dose vaccination base rate	Persons/day	198,600	Internal database of the Department of Disease Control (the largest rate compared to other doses as the second shot currently being the main policy priority)
Booster-dose vaccination base rate	Persons/day	66,200	Internal database of the Department of Disease Control
Vaccine effectiveness against any infection for one-dose vaccination	Unitless	0.17	Adapted from Head and van Elsland [26] and internal database of the Department of Disease Control
Vaccine effectiveness against any infection for two-dose vaccination	Unitless	0.41	Adapted from Head and van Elsland [26] and internal database of the Department of Disease Control
Vaccine effectiveness against any infection for booster-dose vaccination	Unitless	0.65	Adapted from Head and van Elsland [26] and internal database of the Department of Disease Control
Vaccine effectiveness against severe infection for one-dose vaccination	Unitless	0.70	Adapted from Head and van Elsland [26] and internal database of the Department of Disease Control
Vaccine effectiveness against severe infection for two-dose vaccination	Unitless	0.90	Adapted from Head and van Elsland [26] and internal database of the Department of Disease Control
Vaccine effectiveness against severe infection for booster-dose vaccination	Unitless	0.95	Adapted from Head and van Elsland [26] and internal database of the Department of Disease Control
Proportion of intubated cases to existing active infectees	Unitless	0.005	Internal database of the Department of Disease Control (assume half of the proportion of the Delta variant)
Ratio of deaths per existing intubated cases	Unitless	0.03	Internal database of the Department of Disease Control (assume same as the ratio of the Delta variant)
Length of stay for non-intubated cases	Days	10	Internal database of the Department of Disease Control
Length of stay for intubated cases	Days	21	Internal database of the Department of Disease Control
Non-pharmaceutical intervention base effectiveness against any infection	Unitless	See supporting information (Figure S1)	Assume being an exponential function with the incident cases

**Table 2:** The essential formula of the model.

Description	Formula	Note
Rate of change from being susceptible to being exposed	−β × (1 − κ) × (1 − ve) × S × I_1_/P	β = reproduction number/infectious duration, κ = effectiveness of non-pharmaceutical intervention against any infection, ve = effectiveness of vaccine against any infection (varying by vaccine doses), S = susceptible population, I_1_ = non-isolated infectees, P = total population
Rate of change from being susceptible to being non-isolated infectious	−αE	α = 1/incubation period, E = exposed population
Rate of change from being non-isolated infectious to being isolated infectious	−δI_1_	δ = 1/time lag from non-isolation to isolation, I_1_ = non-isolated infectious population
Rate of change from being isolated infectious to being recovered	−ζI_2_	ζ = 1/length of stay; I_2_ = isolated infectious population (varying by severity status)

**Table 3:** Scenarios of interest.

Scenario	Reproduction Number	Vaccination Rate
1	7.5	Base pace
2	7.5	Speedy pace
3	4.3	Base pace
4	4.3	Speedy pace

## Data Availability

Not applicable.

## Supplementary Materials

The following are available online at https://www.mdpi.com/article/10.3390/vaccines10071024/s1, Figure S1: Percentage decrease of contact rate in relation to daily incident cases under the Omicron variant epidemic (base case)—reflecting effectiveness of non-pharmaceutical interventions.
