# Supplementary material for: Forecasted Trends of the New COVID-19 Epidemic Due to the Omicron Variant in Thailand, 2022
Source: Vaccines (Basel). 2022 Jun 27;10(7):1024. doi: 10.3390/vaccines10071024 (PMC9320113; doi:10.3390/vaccines10071024)
Supplement: Supplementary file 1 [file vaccines-10-01024-s001.zip › vaccines-1721793-supplementary.pdf]

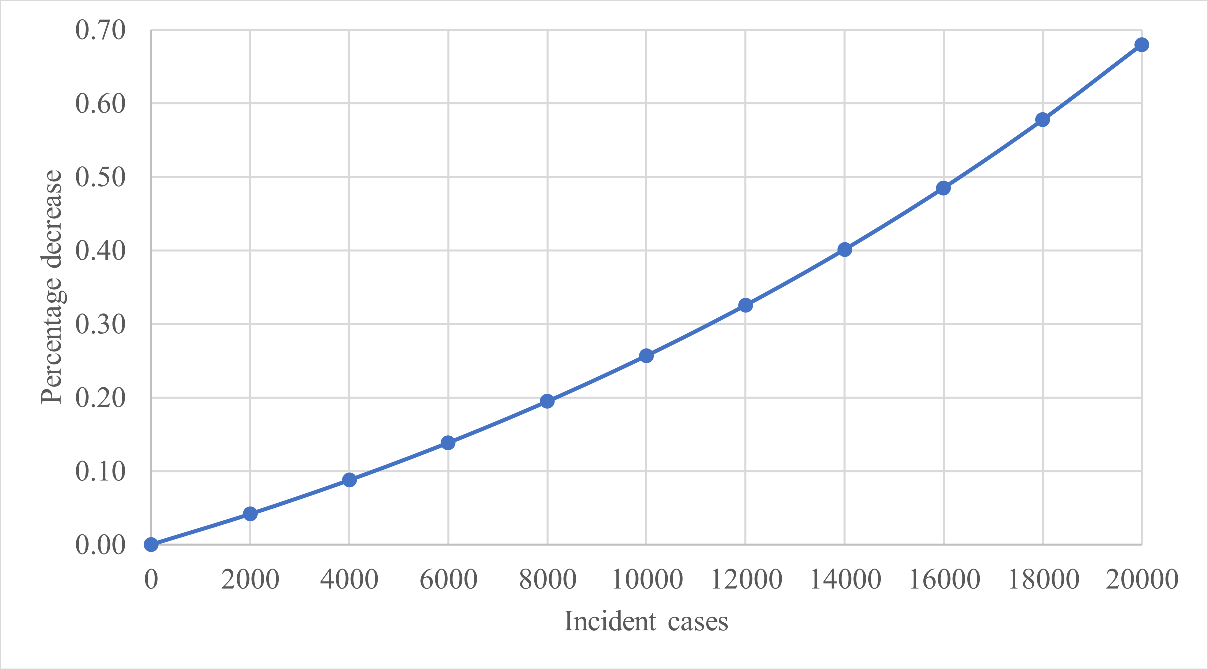

Figure S1. Percentage decrease of contact rate in relation to daily incident cases under the Omicron variant epidemic (base case)—reflecting effectiveness of non-pharmaceutical interventions
